# Influence of stent-induced vessel deformation on hemodynamic feature of bloodstream inside ICA aneurysms

**DOI:** 10.1007/s10237-023-01710-9

**Published:** 2023-03-22

**Authors:** Amir Sabernaeemi, M. Barzegar Gerdroodbary, Sajad Salavatidezfouli, Peiman Valipour

**Affiliations:** 1 Department of Space, Earth and Environment, Chalmers University of Technology, Gothenburg, Sweden; 2 Department of Mechanical Engineering, Babol Noshirvani University of Technology, Babol, Iran; 3 Mathematics Area, MathLab, International School for Advanced Studies (SISSA), Trieste, Italy; 4 Department of Textile Engineering, Clothing and Fashion, Qaemshahr Branch, Islamic Azad University, Qaemshahr, Iran

**Keywords:** Blood Rheology, CFD, Polymer Stent, Cerebral aneurysm, Viscous flow, Lateral ICA

## Abstract

One of the effective treatment options for intracranial aneurysms is stent-assisted coiling. Though, previous works have demonstrated that stent usage would result in the deformation of the local vasculature. The effect of simple stent on the blood hemodynamics is still uncertain. In this work, hemodynamic features of the blood stream on four different ICA aneurysm with/without interventional are investigated. To estimate the relative impacts of vessel deformation, four distinctive ICA aneurysm is simulated by the one-way FSI technique. Four hemodynamic factors of aneurysm blood velocity, wall pressure and WSS are compared in the peak systolic stage to disclose the impact of defamation by the stent in two conditions. The stent usage would decrease almost all of the mentioned parameters, except for OSI. Stenting reduces neck inflow rate, while the effect of interventional was not consistent among the aneurysms. The deformation of an aneurysm has a strong influence on the hemodynamics of an aneurysm. This outcome is ignored by most of the preceding investigations, which focused on the pre-interventional state for studying the relationship between hemodynamics and stents. Present results show that the application of stent without coiling would improve most hemodynamic factors, especially when the deformation of the aneurysm is high enough.

## Introduction

### IA and it pathophysiology

The effects of hemodynamic forces on the wall of the intracranial artery may result in a pathological stretching of the vessel wall, and a deformed vessel is known as an Intracranial Aneurysm (IA). The development of IA is relevant to many pathophysiological factors, not only the hemodynamic ones, e.g., endothelial function, anatomic variations (certain areas in the Circle of Willis) are more vulnerable (Liu 2020). The main characteristic of this extension is the weak structural strength of this region which may result in rupture and haemorrhage. In fact, the shape of the stent can also significantly influence focal hemodynamics in intracranial arteries (Liu 2022). Although several investigations have been done to detect the sign of aneurysm formation, detection of unruptured aneurysms in the initial stage is not possible yet (Ashkezari 2021; Sforza et al. 2009; Martu et al. 2014). Surgical researches have extensively used for the treatment of different patients (Zhang et al 2022; Zhao et al 2022; Hu et al 2022). Most of these researches use CT images (Jin et al 2022; Ban et al 2022) and data processing for analysis of biomedical data (Lu et al 2023; Qin et al. 2022; Yang et al 2022). Artificial techniques have been used extensively for biomedical investigations (Liu et al 2022a, b; Lyu et al. 2023; Jin et al. 2015). Different works also used theoretical method for the clinical data (Hao et al 2022; Zhou et al 2022; Xue et al 2022; Yang et al 2022; Zhang et al 2022). Applications of these computer methods are conventional in biomedical science and technology (Chen et al. 2021; Lin et al 2022; Zheng et al 2022; Li et al. 2022; Wang et al 2023; Sun et al 2022).

### Hemodynamic parameter relevant to the development and rupture of IA

The primary hemodynamic factors of Oscillatory shear index (OSI), Wall shear stress (WSS) and Relative residence time (RRT) are introduced for the comparison of the different ICA aneurysms (Xiao-Yong et al. 2022; Fung, 1993). Besides, some critical values are also presented for recognising high-risk regions of the aneurysm. Nevertheless, numerous of the experimental outcomes attained via this technique are not consistent with actual pathophysiological indicators, which has mystified clinicians and researchers (Razavi et al. 2011).

An outer elastic layer has little supporting tissue nearby the artery with thin medial elastin, and this is the primary insufficiency of the wall of the cerebral artery (Hariri et al. 2023). Besides, an unbalanced anatomical configuration at its bifurcation apex is another deficiency of the artery wall. Due to these features, continuous action of blood flow could easily result in local structural changes in the wall of the cerebral artery. External factors and genetic susceptibility are known as the primary source for the formation of IAs, and this process is complex and diverse. Based on the published articles, the growth and rupture of IAs happen under the impacts of WSS (Li et al. 2022; Lyu and Wang 2023; Mansouri et al. 2022b; Sadeh et al. 2023; Sheidani et al. 2023; Yang et al. 2023; Zhang et al. 2021). Preceding works found that aneurysm growth initiates due to the high value of WSS and OSI, while rupture occurs in a low WSS. The interaction between vascular physiological changes and haemodynamics indicates that WSS induces vascular endothelial irritation, and upregulates the expression of adhesion cytokines and molecules on the surface of the arterial wall. Hence, the permeability of the vascular lumen is increased and this also results in a high residence time of blood on the arterial wall. Therefore, the migration of leukocytes to the wall is promoted and consequently, a large number of matrix metalloproteinase are produced.

### Application of CAD in intracranial arteries and IA

The research on this topic has been significantly improved by the invention of magnetic resonance angiography (MRA) and computed tomography angiography (CIA). In fact, these techniques enable researchers to access the main geometrical aspects of the aneurysm and use them for further investigations (Coady et al. 1997; Abdehkakha et al. 2021). They analyzed blood hemodynamics by this technique to find the main source of the aneurysm growth. Meanwhile, the analysis of the high-risk region of the aneurysm becomes possible with these techniques (Lobato and Puech-Leao 1998; Eleftriades 2002; Mirzaei Poueinak et al. 2023).

One of the main advantages of access the real three-dimensional geometry of IAs is computational modelling (Pape et al. 2007; Celi and Berti 2014). The progress in the computational fluid dynamic significantly helps scientists to access diverse hemodynamic factors for evaluating of the aneurysm rupture risk (Fillinger et al. 2003; Vande Geest et al. 2006). In CFD methodology, medical image processing, vascular wall reconstruction, and mesh generation technology are combined, and the finite element technique is used to compute haemodynamic parameters (Gallo, et al. 2012). Lastly, the spatial and temporal distribution characteristics of blood flow are obtained. Indeed, a fundamental cause in the creation of intracranial aneurysms (IAs) is blood flow (Martu et al. 2016). The inflammatory dispersal cells are defined by their features, and the inflammatory reaction of endothelial cells, which are the primary boundary with blood flow, is determined via these factors (Xiao-Yong et al. 2021a, b). A thorough endothelial cell layer could guarantee the permanence of the vascular lumen by controlling the distribution of anti-inflammatory and anti-thrombotic features (Xiao-Yong et al. 2022).

### Relevant CFD studies and limitations (i.e., research gaps)

Analysing the impacts of the endovascular technique on the risk reduction of the aneurysm rupture is crucial. Usage a stent as the leading conventional endovascular technique for the treatment of the saccular aneurysm has been investigated. Currently, there are two different stent options employed in neurointerventional radiology namely, flow diverter stents and regular intracranial stents. These two stent types have different mesh densities and radial forces. In fact, flow diverter stents have a lower radial force than intracranial support stents, and listed papers did not report significant safety concerns with CFD analysis (Sadeh et al. 2023; Mutlu, et al. 2020). The main application of the stent without coiling is to deform the artery wall to reduce the entrance of the mass blood flow into the aneurysm. Although usage of the stent is to keep the coiling gel inside the aneurysm, it could also be used as an independent technique for the reduction of the aneurysm protection, especially when the risk of the rupture is not high (Mirzaei Poueinak et al. 2023).

### The aim of this study

In the present work, the influences of the stent on the hemodynamic aspects of four different ICA aneurysms have been investigated. This research has focused on the hemodynamic characteristics of WSS and OSI in two stages of aneurysm deformation due to the stent application. Computational fluid dynamic (CFD) is used to model of blood flow inside the aneurysm and calculating shear stress on the aneurysm wall. Comparisons of blood velocity and stream are also presented to reveal the influence of aneurysm deformation on the blood and its impact on the wall of the vessel.

## Materials and methods

### Aneurysm selection

**Fig. 1:**
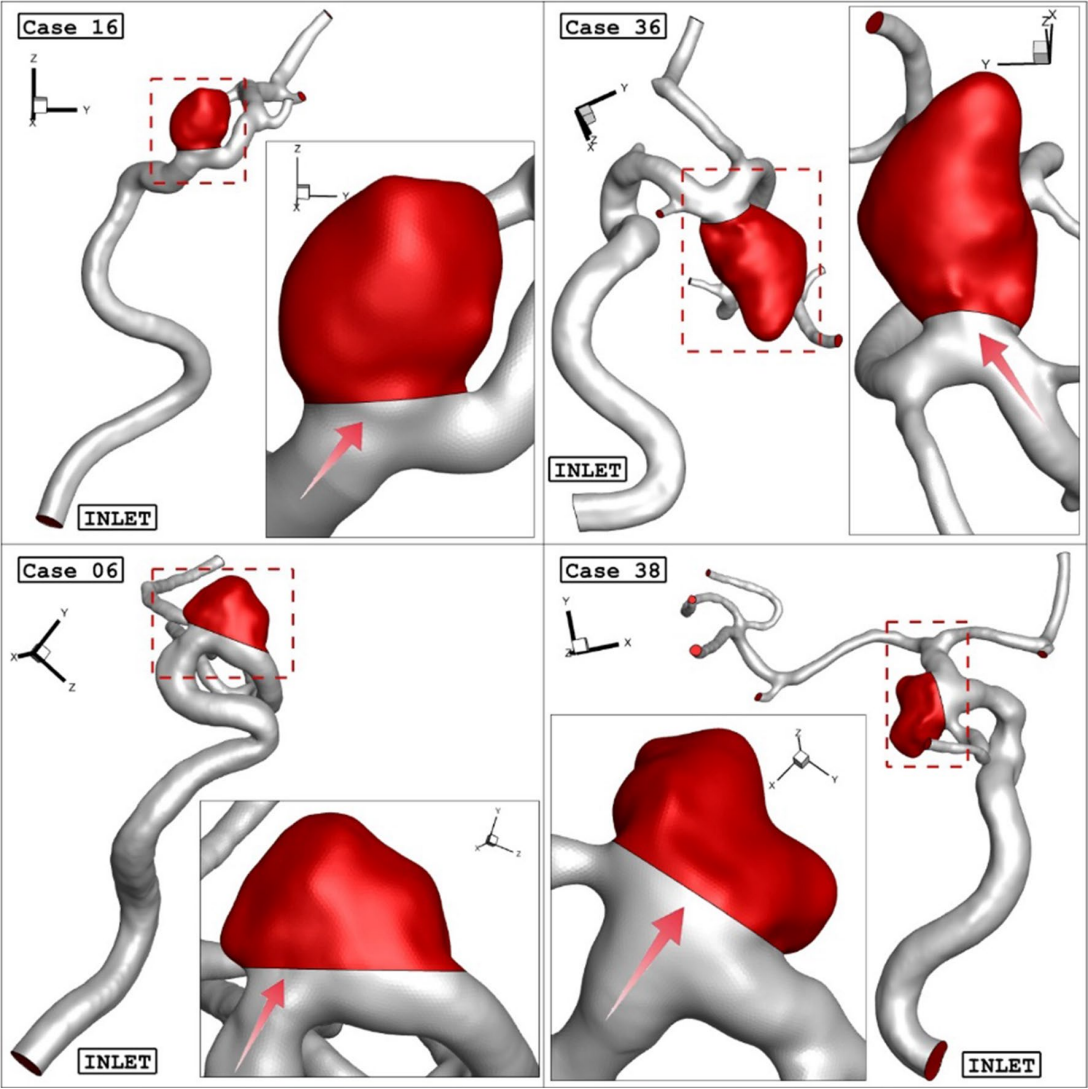
Geometry of selected aneurysm with blood flow direction

After evaluating of more than 30 ICA aneurysms, the geometries (.stl file) of four distinctive aneurysms are chosen from the Aneurisk website (AneuriskWeb project website 2012). The aneurysms are sorted by the size of the sac section area at the ostium. To investigate the influence of the deformations, the angle of parent vessel orientation with normal ostium plane is high in the selected aneurysm. It is assumed that the presence of a stent reduces this angle and, consequently, limits the blood flow rate into the sac region. Figure 1 displays the schematic of four selected ICA aneurysms. The details of the chosen ICA geometries are presented in Table 1.

**Table 1:** Specifications of selected aneurysms

Case ID	Ostium section area (mm^2^)	Neck vessel angle (degree)	Sex
16	21.2	50.9	Female (HCT = 0.40)
36	26.86	41.8	Male (HCT = 0.45)
06	34.9	31.4	Female (HCT = 0.40)
38	38.4	45.4	Female (HCT = 0.40)

### Computational technique

**Fig. 2:**
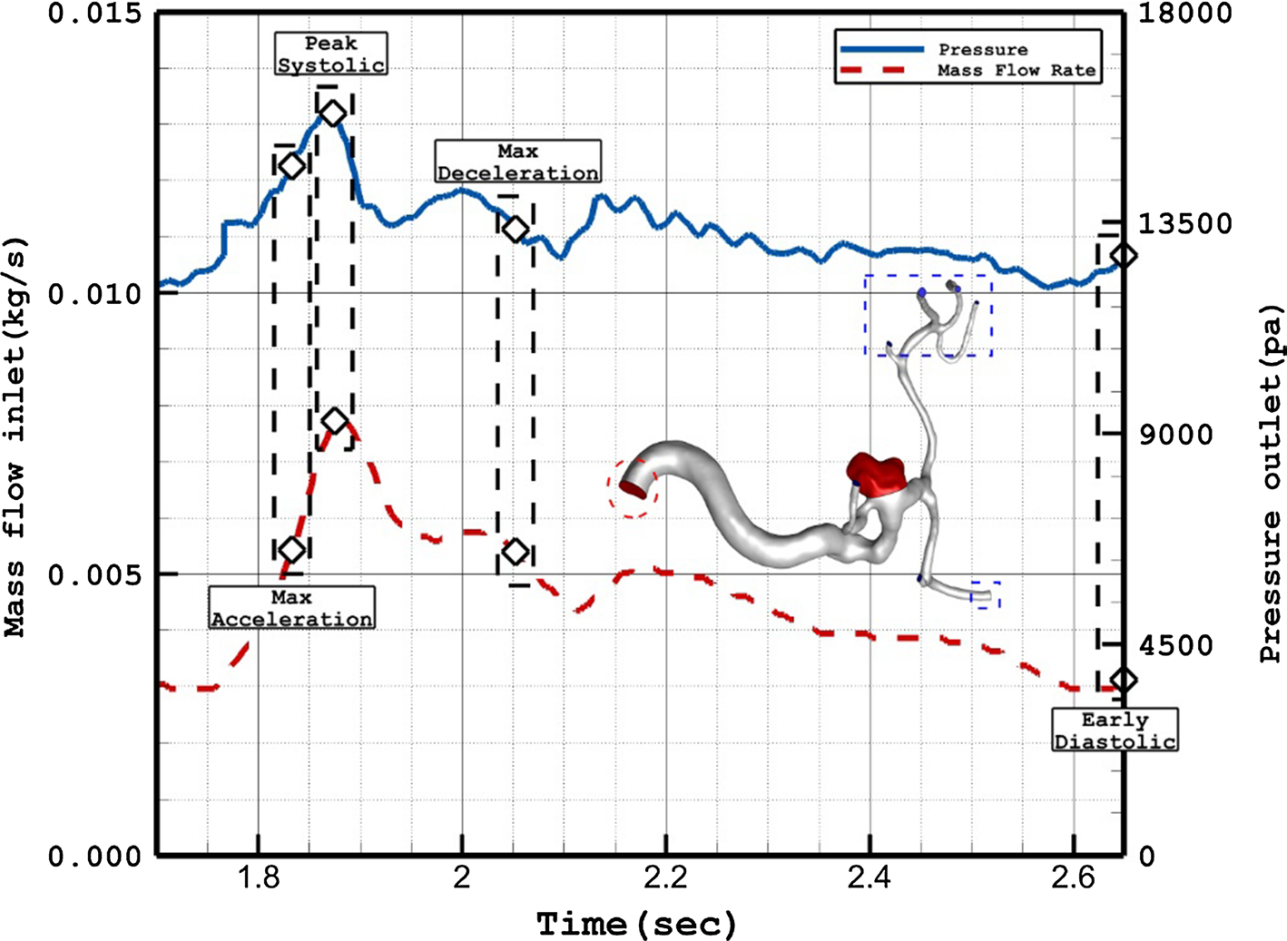
Applied mass and pressure profile at inlet and outlets

The simulation of the blood stream inside the saccular aneurysm is done by solving the transient RANS equations while blood is assumed non-Newtonian and incompressible. One-way FSI is used for the interaction of the blood and vessel in which the blood force is affected on the aneurysm as an external force (Samuel et al. 2019; Chen and Cai 2021; Xu et al. 2018). Since blood flow inside the aneurysm is pulsatile, the mass flow rate at the inlet and pressure value at the outlet are applied by the displayed pattern in Fig. 2 (Malvè et al. 2014). Inlet profile to use coarser grid, standard wall function, recording specific results, and using Paraview for post processing is explained in detail by (Mansouri et al. 2021 and Verma, et al. 2021). In the present study, three cardiac cycles are simulated and the length of each cardiac cycle is about 0.875 s and time step is 8.75 e-4 s.

**Fig. 3:**
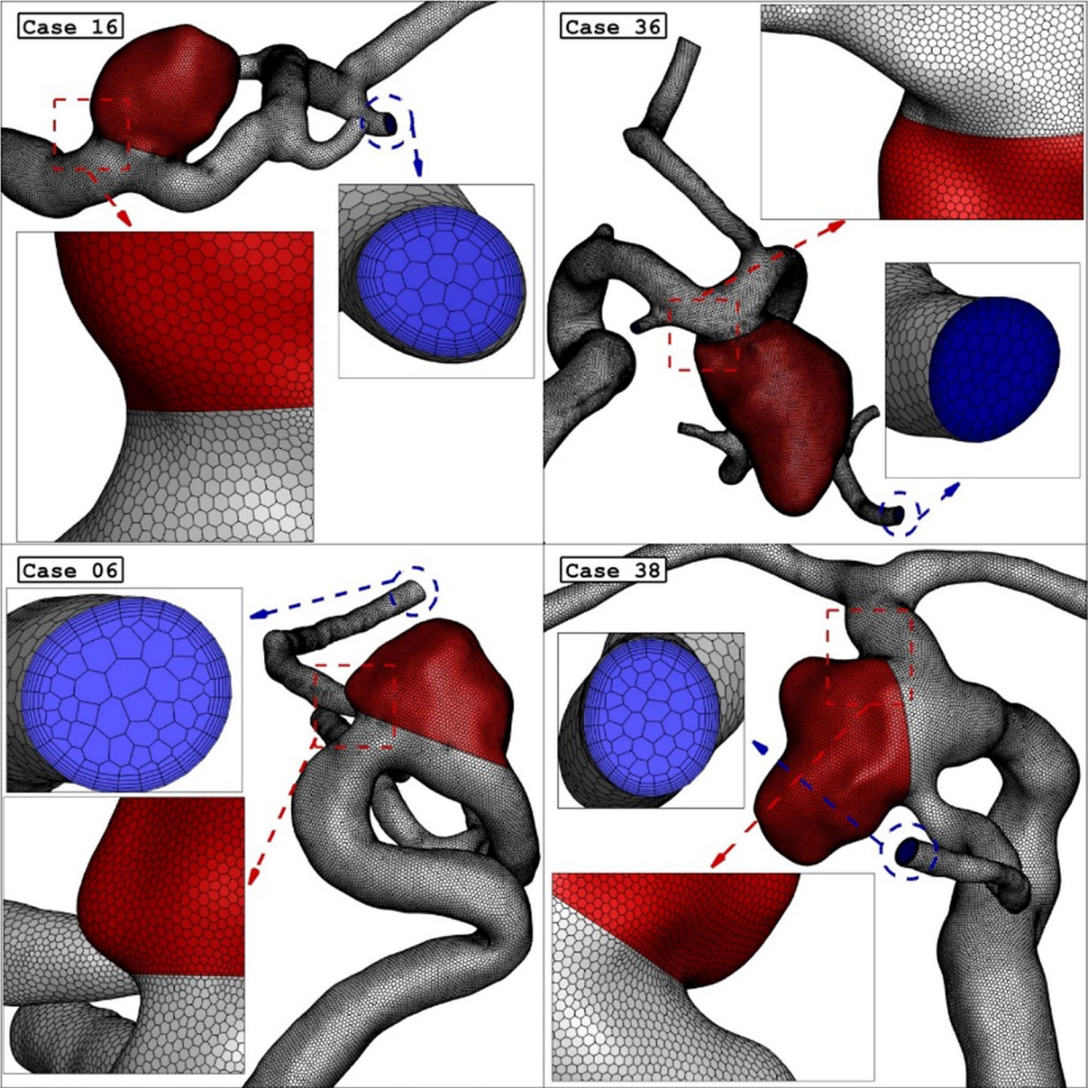
Grid generation for 4 different ICA cases (Main Models: Before Deformation)

The blood viscosity is calculated by the Casson model Razavi et al. (2011). Non-Newtonian rheological model plays an important role in improving the accuracy of WSS in aneurysms since Newtonian rheological model can cause considerable inaccuracy (> 10% in WSS) in low-WSS areas, not only in extracranial arteries such as coronary arteries (Liu et al. 2018) but also in intracranial arteries (Liu et al. 2021). However, different non-Newtonian models, e.g., Casson model and Carreau-Yasuda model, behave differently in low-shear range. Therefore, different non-Newtonian models can be applied in future studies for further validation of the findings. In this model, the effect of hematocrit is also used for the estimation of the viscosity. The generated grid for the selected aneurysm are demonstrated in Fig. 3. The size of the grid near the vessel is smaller than other regions due to the importance of this region for the estimation of the WSS and OSI factors (Boccadifuoco et al. 2018; Aristotelis et al. 2008).

**Fig. 4:**
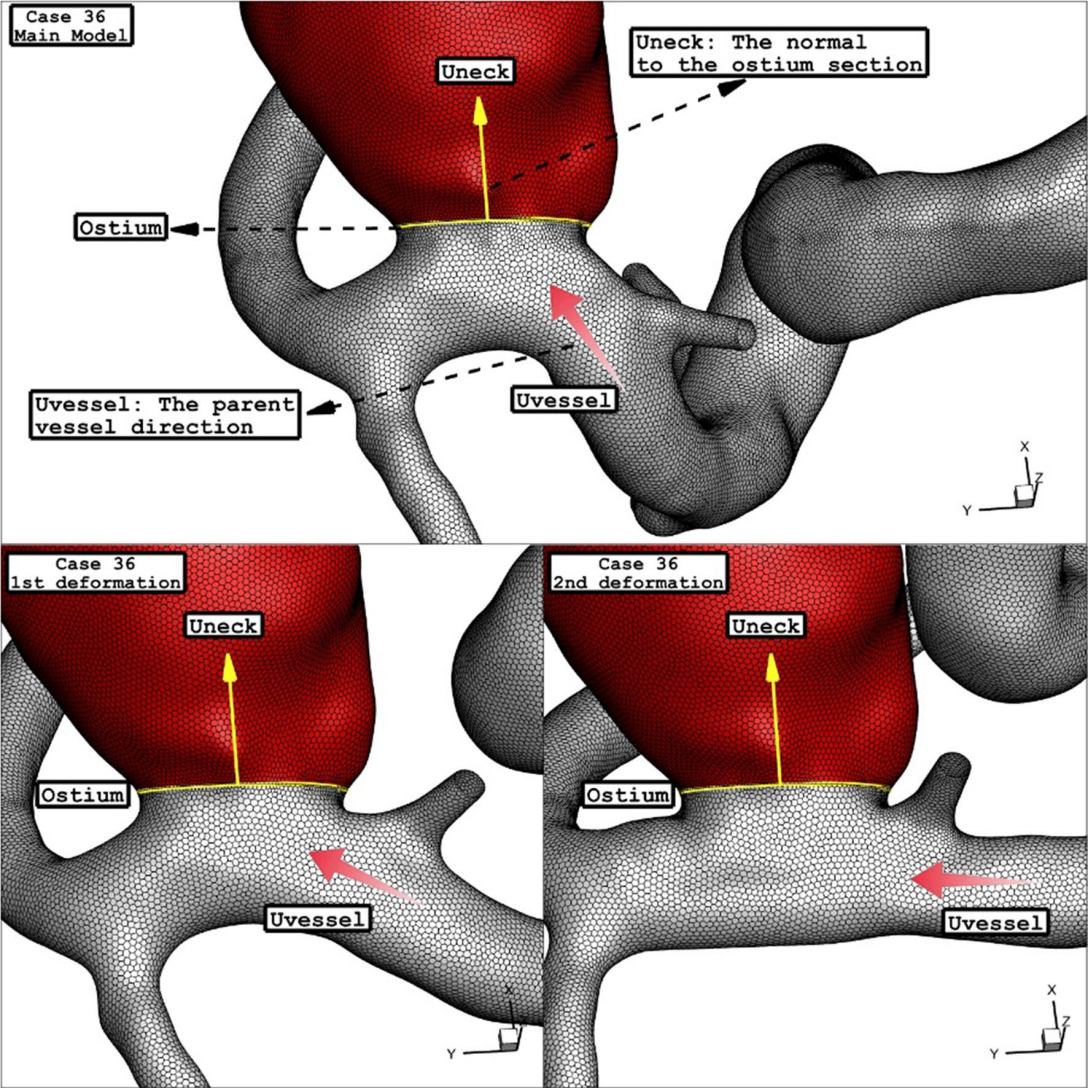
Effects of deformation on vessel and grids

As demonstrated in Fig. 3, the boundary layer is applied for the produced grid near the vessel to improve the accuracy and precision of the results (Zan-Hui et al. 2023). The size of grid near the vessel and aneurysm a wall is high since haemodynamic factor is calculated in this section. Mesh density near wall is higher and the number of boundary layers for aneurysm wall is 9. Skewness of produced grid is within acceptable limit. The inflow boundary condition is chosen regardless of the largest grid spacing size, it leads to spurious pressure production in the domain (Mansouri et al. 2022a) To preserve computational time, size of grid in the centre of vessel is higher than other segments since less variation happens in these regions. For using the deformations, the sac section area (red volume) is split. To perform grid independency, four different grids are examined, and the results of the velocity for the specific model (case 16) are demonstrated in Table 2. As presented in this table, the average velocity on the ostium section is compared for different produced grids. It is noticed that the value of this factor does not change meaningful when a model with fine grid is replaced by a very fine one. Therefore, the grid size of this model is applied to other aneurysms. Figure 4 presents more details about the grid production to the specific model after deformation.

**Table 2:** Details of used grids

	Cells	Average blood velocity at inlet of sac (maximum acceleration)	Average blood velocity at inlet of sac (Peak systolic)
Coarse	1,022,000	0.241	0.363
Medium	1,820,000	0.249	0.389
Fine	2,424,000	0.255	0.396
Very fine	2,948,000	0.258	0.397

Since the worst-case scenario is related to the peak systolic stage (2120 step), this study reports WSS and average pressure on the vessel and mean velocity inside the sac for this stage. Meanwhile, the OSI value is calculated at the end of the third cardiac cycle (3000 steps) [22, 23, 24, and 25].

### Deformations of the aneurysm (impact of stent)

**Fig. 5:**
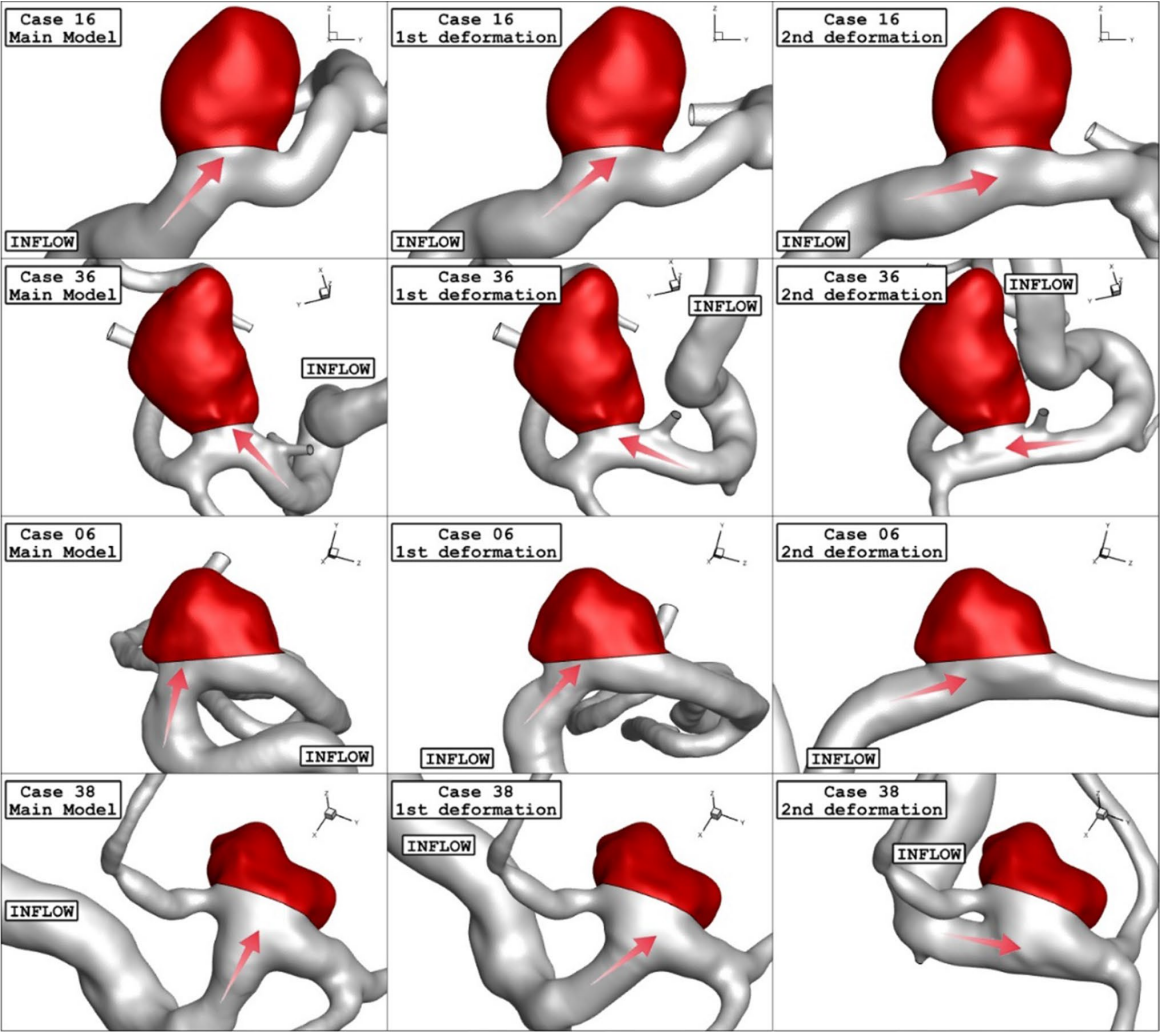
Geometry of ICA models and their deformations

As explained in the previous sections, the main attitude of the present work is to investigate the role of the stent on the hemodynamics of the blood stream inside the aneurysm. In the current study, the two stages of interventions are assumed based on the neck vessel angle mentioned in Table 1 for the deformed aneurysm. Figure 5 illustrates the 1st and 2nd stages of the deformed aneurysms as well as primary model of these four chosen lateral ICA aneurysms. It is noticed that the angle of the parent vessel orientation with the normal vector of the ostium plane is maximum, in which less blood stream will enter to sac section (Fig. 4).

## Results and discussion

**Fig. 6:**
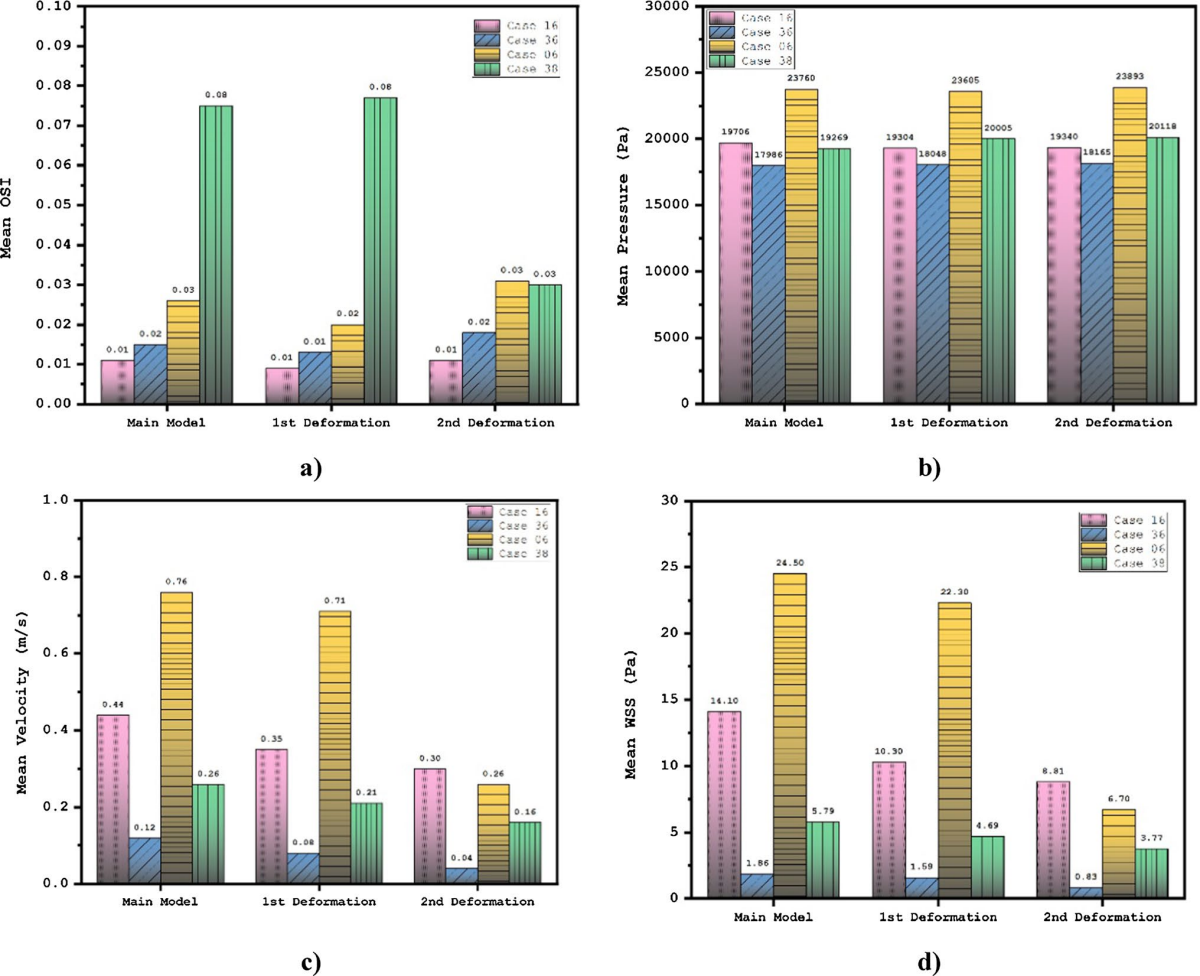
Deformation effects on mean values of WSS, OSI, sac wall pressure, and sac velocity

**Fig. 7:**
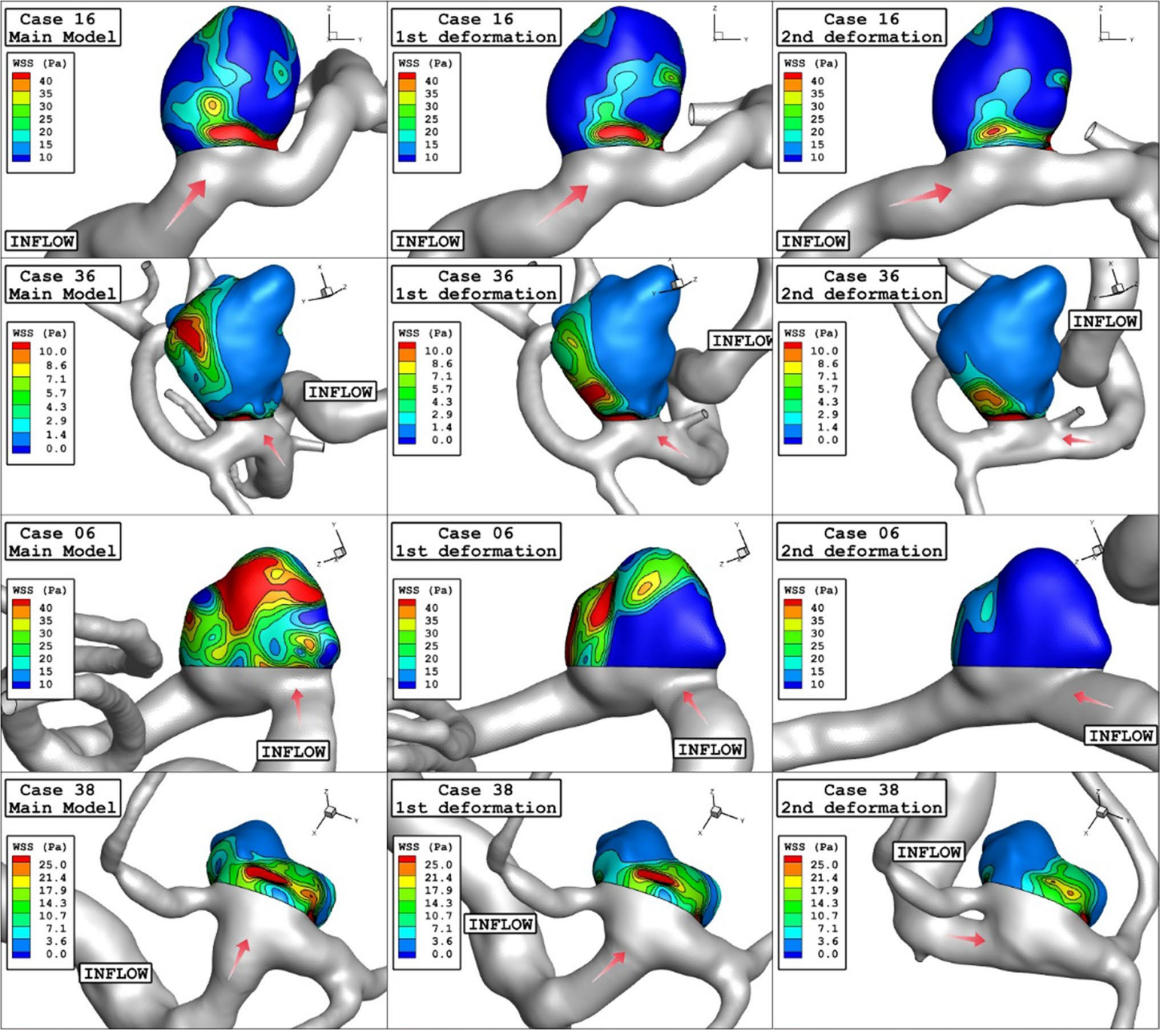
WSS contours (Peak systolic) in different neck vessel angle

The impacts of aneurysm deformation on the main hemodynamic factors of mean WSS, OSI, pressure and velocity are demonstrated in Table 3. To compare the results, Fig. 6 plots the variation of these factors in different stages of deformations. Comparison of results shows that the impact of deformation for those cases with higher mean WSS is more substantial than other cases. The variation of mean OSI indicates that this factor does not change substantial except for cases with high mean OSI. The impact of deformation on the mean pressure is limited, while mean velocity is significantly reduced when the deformation angle is high enough. The contour of the WSS on the aneurysm wall would demonstrate the main changes due to the deformation of the aneurysm. Evaluation of WSS contour (Fig. 7) indicates that the value of maximum WSS on the sac wall substantially decreases after 2nd deformation. Besides, the region with high WSS is limited when deformation is progressed.

**Table 3:** Results of deformation on hemodynamic charactristics

	Model number	Deformation	WSS_mean (Pa)	OSI_mean	Wall pressure_mean (Pa)	Aneurysm Velocity_mean (m/s)
Case16	1	Main case	14.1	0.011	19,706	0.44
	2	1st deformation	10.3	0.009	19,304	0.35
	3	2nd deformation	8.81	0.011	19,340	0.30
Case36	1	Main case	1.86	0.015	17,986	0.12
	2	1st deformation	1.59	0.013	18,048	0.08
	3	2nd deformation	0.83	0.018	18,165	0.04
Case06	1	Main case	24.5	0.026	23,760	0.76
	2	1st deformation	22.3	0.020	23,605	0.71
	3	2nd deformation	6.7	0.031	23,893	0.26
Case38	1	Main case	5.79	0.075	19,269	0.26
	2	1st deformation	4.69	0.077	20,005	0.21
	3	2nd deformation	3.77	0.030	20,118	0.16

**Fig. 8:**
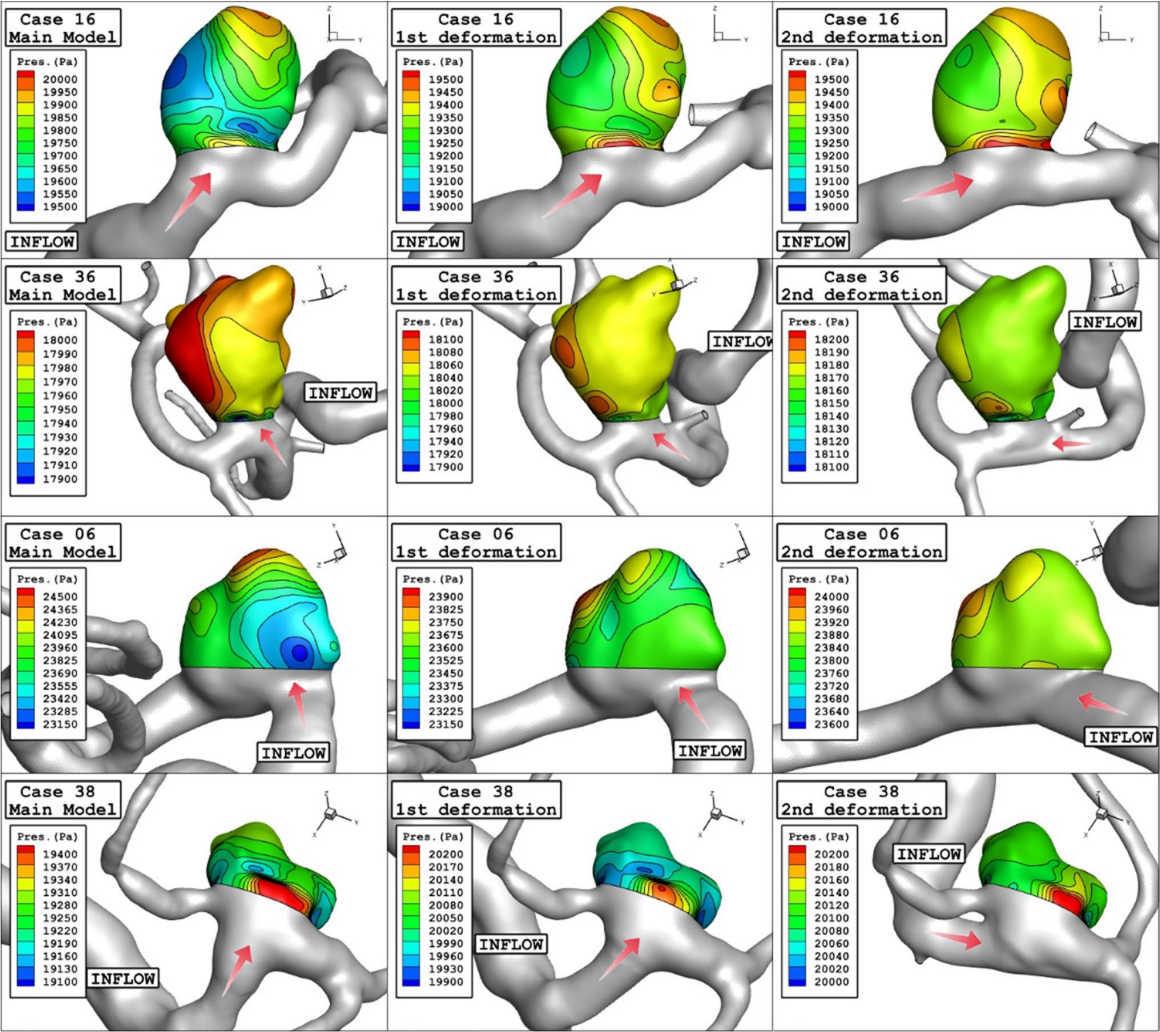
Wall pressure contours (Peak systolic) in different neck vessel angle

Figure 8 illustrates the variation of the pressure contour on the aneurysm wall for a selected aneurysm in different stages of deformations. The value of maximum pressure does not decrease substantially after the deformation, while the location of high-pressure regions varies owing to the deformation. It is mainly due to the change of blood inflow direction after deformation of the aneurysm.

**Fig. 9:**
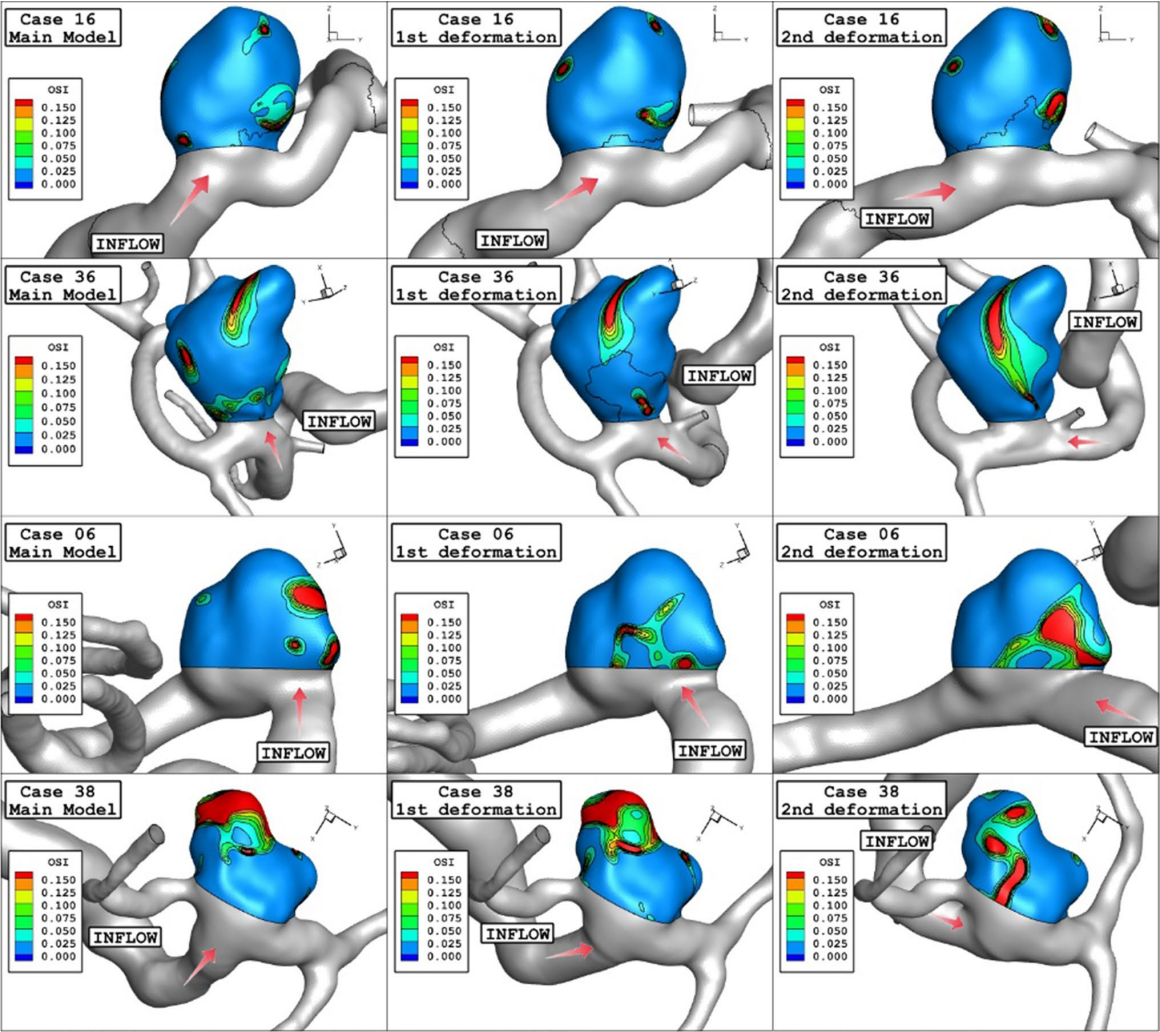
OSI contours (Early diastolic) in different neck vessel angle

**Fig. 10:**
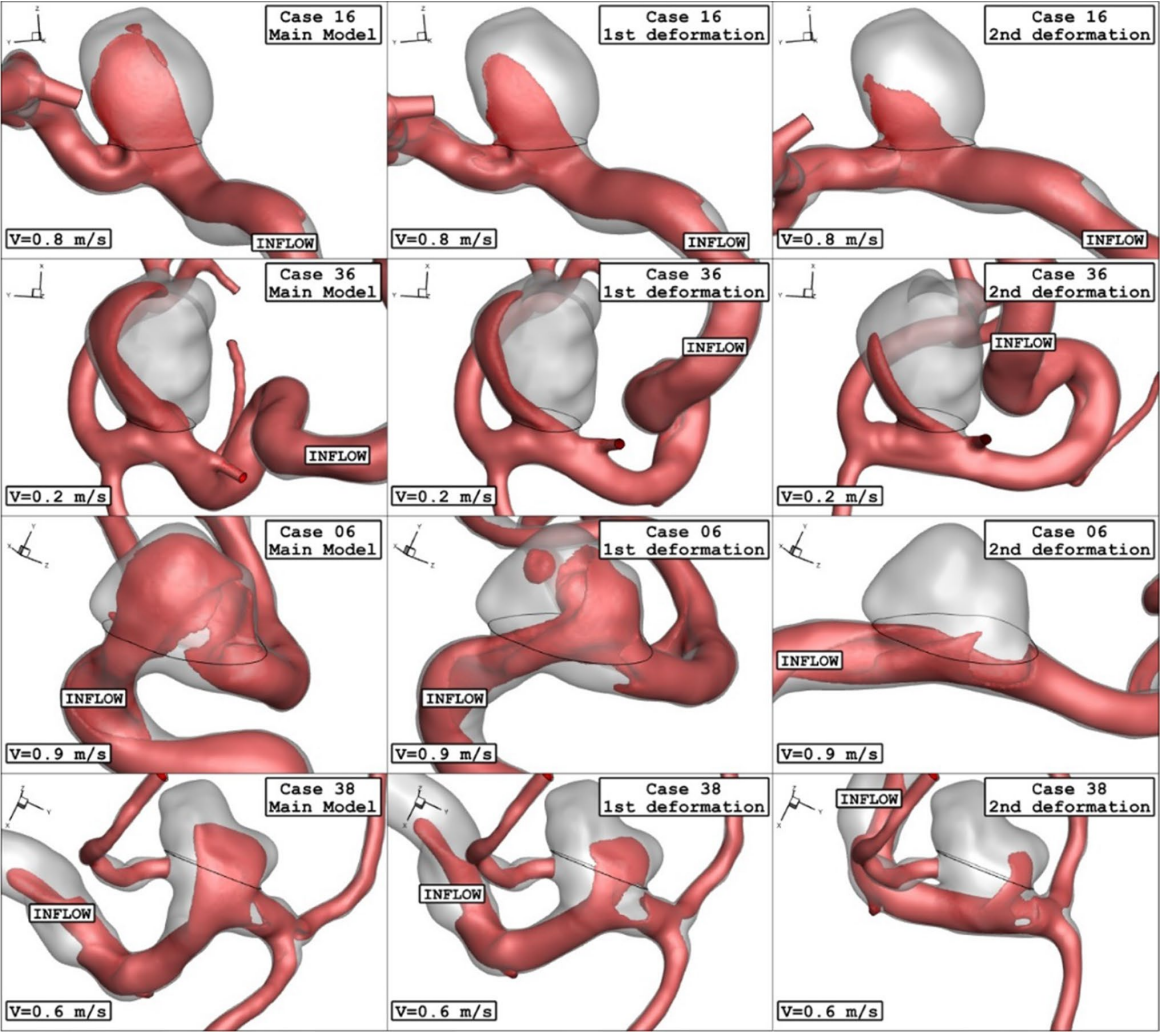
Iso-Surface (velocity at peak systolic) in different neck vessel angle

Comparison of OSI contour for original and deformed aneurysms are displayed in Fig. 9 at the early diastolic stage. The change in the OSI value does not follow specific patterns. In fact, deformation of the aneurysm would decrease the inlet blood velocity, and this enables high-velocity change inside the aneurysm. Therefore, OSI might increase on the sac surface due to deformations. For a better comparison of the deformation effects, Fig. 10 displays iso-surfaces of averaged velocity field to compare aneurysm inflow jet before and after deformation. A comparison of these contours shows that the blood flow is considerably limited because of aneurysm deformation. The high-velocity region is limited to the ostium section when the angle of deformation is high (2nd stage). These blood feature also demonstrates how the flow results in local WSS patterns.

**Fig. 11:**
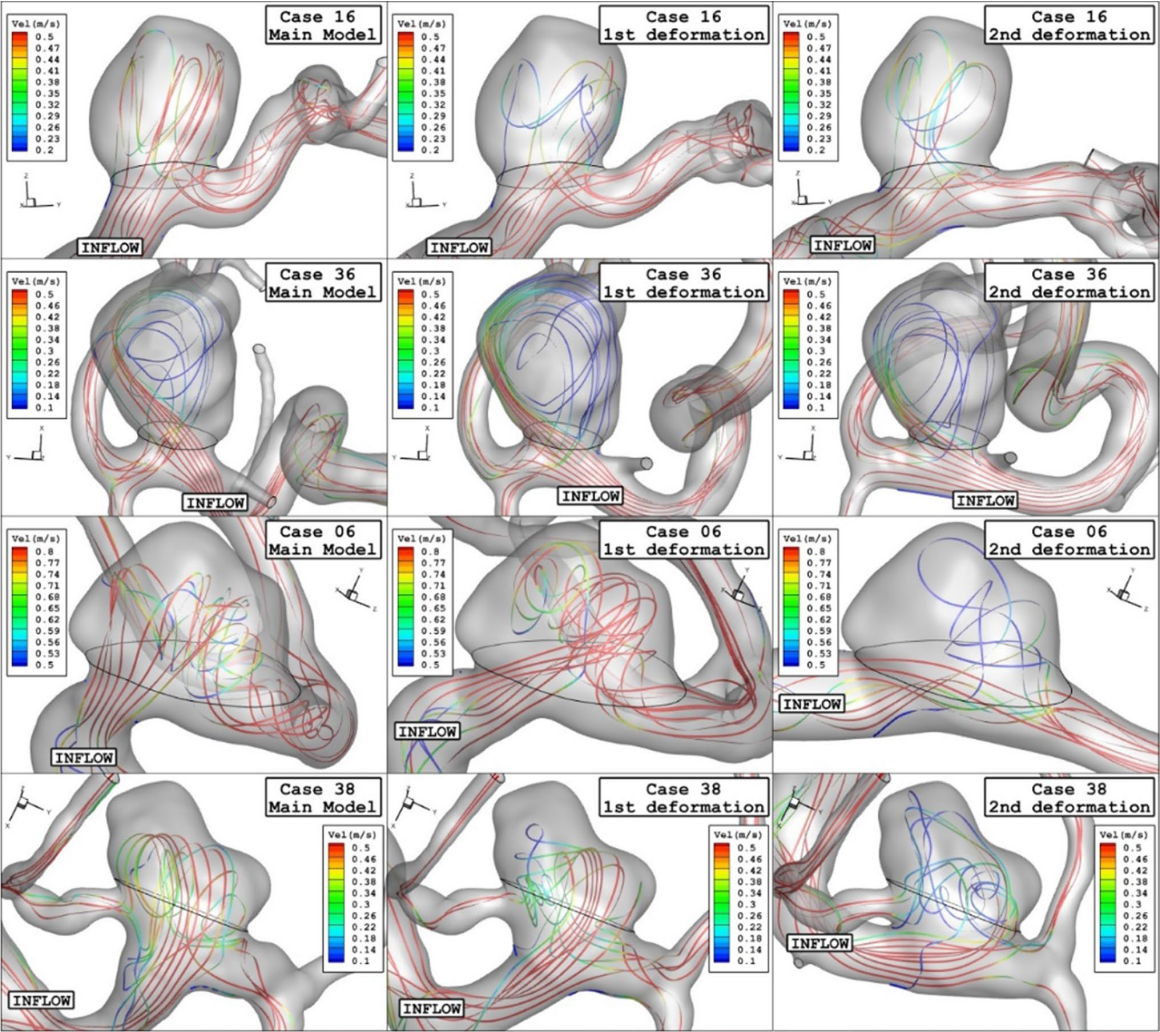
Streamlines (velocity at peak systolic) in different neck vessel angle

Figure 11 demonstrates the flow stream inside the aneurysm with/without deformation at the peak systolic stage. The blood stream is coloured by the velocity value to disclose the impact of deformation on the value of the blood stream inside the sac section. These contours also show that structure of the blood flow change by the aneurysm deformation, and its effects directly related to the angle of deformation. It is found that aneurysm deformation not only limits the flow pattern inside the sac but also alters the velocity value which results in the low WSS inside the sac.

## Conclusion

In this study, the impacts of the stent on the flow structure of the four ICA aneurysms are comprehensively investigated. The primary attention of this research is to visualize blood flow and compared hemodynamic characteristics of ICA aneurysms before and after aneurysm deformation. Computational Fluid dynamic is applied to simulate the blood stream inside the aneurysm and calculate hemodynamic factors, i.e. WSS, pressure and OSI on the aneurysm wall. The stent effect on two-stages of deformation is disclosed and explained in this work. Attained results indicate that deformation of the aneurysm considerably decreases the WSS on the aneurysm wall due to limited blood entrance. However, the value of OSI does not change in deformed aneurysms. Pressure contour on the aneurysm wall also indicates that the value of this factor does not alter considerably while its location varies by deformation.

## Data Availability

All data generated or analysed during this study are included in this published article.
